# Sustainable UV approaches backed by one step extraction procedure for quantifying the newly released mirabegron and silodosin mixture in urine

**DOI:** 10.1038/s41598-025-13989-y

**Published:** 2025-08-14

**Authors:** Ahmed R. Mohamed, Sara El‑Hanboushy, Eman Darweish

**Affiliations:** 1 Pharmaceutical Analytical Chemistry Department, Faculty of Pharmacy, Egyptian Russian University, Badr City, Cairo 11829 Egypt; 2 Pharmaceutical Chemistry Department, Faculty of Pharmacy, Future University in Egypt, Cairo, 11835 Egypt

**Keywords:** Mirabegron, Silodosin, SALLE, Fourier self-deconvolution, Induced dual-wavelength, Chemistry, Analytical chemistry

## Abstract

Mirabegron (MIR) and silodosin (SIL) have recently been combined in a single pill to significantly enhance the effectiveness of treating detrusor hyperactivity with impaired contractility (DHIC), leading to appreciable improvements in symptoms associated with overactive bladder. Additionally, this combination effectively manages lower ureteric stones and improves patient outcomes with no significant side effects, especially in elderly patients. Accordingly, this study introduces two UV techniques for analyzing MIR and SIL in their mixtures (pure and commercial mixtures). These techniques were backed by a one-step salting-out liquid/liquid extraction (SALLE) procedure for quantifying MIR and SIL in urine samples without matrix interference. The proposed UV techniques succeeded in resolving the superimposed MIR’s and SIL’s UV spectra by employing straightforward mathematical filtration. The UV techniques were Fourier self-deconvolution (FSD) and induced dual-wavelength (IDW) techniques, with linearities of (50–350) µg/mL for MIR and (5–100) µg/mL for SIL. The applied techniques were verified following the International Council for Harmonisation (ICH) directives and were statistically evaluated against the published technique, with no noteworthy differences found. The applied techniques’ practicality (blueness), whiteness, and greenness were appraised utilizing various metrics. Per the preceding, the applied approaches have been proven to be sustainable, delicate, and appropriate for quality control (QC) testing. Also, backing the applied approaches with the SALLE procedure enables precise monitoring of MIR and SIL in miscellaneous biological fluids with excellent recoveries, presenting an inventive approach for further bioanalytical applications.

## Introduction

Elderly people frequently suffer from overactive bladder syndrome (OAB) and benign prostatic hyperplasia (BPH), which can cause a lower quality of life and worsen lower urinary tract symptoms (LUTS)^1,2^. Clinically, BPH manifests as a spectrum of LUTS, categorized as obstructive (e.g., weak stream, urinary hesitancy, straining), irritative (e.g., frequency, nocturia, urgency), and post-void (e.g., dribbling, incomplete emptying)^1^. Urinary urges that are strong, frequent, and uncomfortable are common in overactive bladders^3,4^. Urinary retention is more likely to occur with age and worsening symptoms. Other conditions, such as prostate or bladder cancer, kidney stones, and an overactive bladder, can also cause symptoms of BPH^3,4^.

**Fig. 1:**
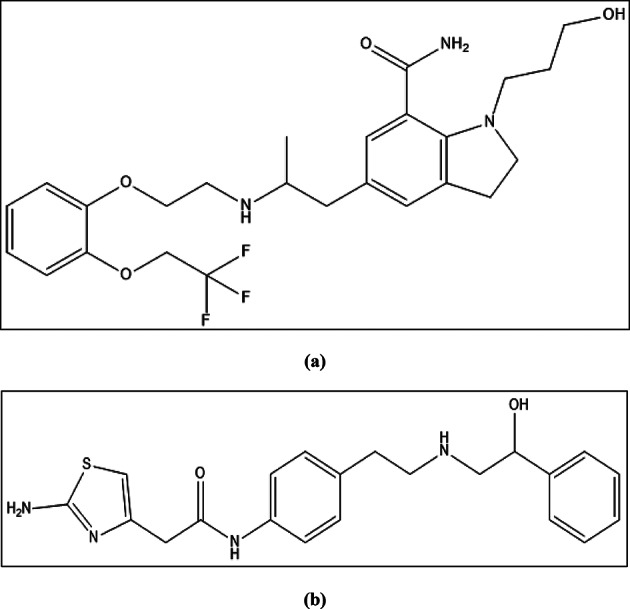
Chemical structures of (**a**) SIL and (**b**) MIR.

SIL (Fig. 1a) acts as an alpha-1 (α_1_) adrenergic receptor antagonist for managing the signs of BPH. SIL’s marked uroselectivity, stemming from its strong affinity for α_1A_ receptors, makes it a preferred α-blocker for treating LUTS and BPH compared to less selective agents^5^. MIR (Fig. 1b), the first β_3_-adrenoceptor agonist to reach clinical practice, is FDA-approved for treating OAB. MIR is an efficient therapy choice for older people with urodynamic detrusor overactivity and DHIC^6^. Individuals over sixty years of age who use daily drugs for several chronic diseases are susceptible to overmedication and other polypharmacy consequences, which can include a variety of adverse effects^7^. By combining MIR and SIL, DHIC can be treated more effectively, and the overactive bladder symptom score (OABSS) can also be improved, with no significant increase in drug-related adverse effects^8^. Furthermore, MIR and SIL offer effective treatments for removing lower ureteric stones in addition to enhancing stone expulsion and shortening the duration of the process^9^.

Hence, specific analytical procedures had to be developed to quantitatively assess the combination of raw materials and medicinal products. After conducting a thorough analysis of the literature on the selected medications, only two chromatographic papers^10,11^ and one UV-spectrophotometric paper^12^ were found, describing the quantification of MIR and SIL in their synthetic mixtures. Accordingly, we introduce two straightforward UV techniques for analyzing MIR and SIL in their pure and final commercial mixtures. Furthermore, we backed the proposed techniques with a one-step SALLE procedure for quantifying MIR and SIL in urine samples without matrix interference.

The SALLE technique utilizes an organic solvent that is miscible with water, such as methanol, as the extraction solvent. Analytes transfer to the organic layer through phase separation induced by the addition of concentrated salts. This method is easier to understand than liquid/liquid extraction (LLE) and does not require prolonged mechanical shaking due to the miscibility of the two stages (phases)^13^. SALLE is considered to be a valuable technique or procedure for the preparation of drug samples for quantification purposes. It has proven its effectiveness in meticulously extracting a wide range of drug classes from complex matrices, such as blood or urine, with minimal losses caused by matrix interference and high sensitivity^13^.

Green analytical chemistry (GAC) aims to reduce the impact of laboratory research methods on the environment. In the fast-growing pharmaceutical business, understanding the significance of green-and-white analytical chemistry has become crucial. Using our approaches is a green alternative to analyzing pharmaceutical compounds (MIR and SIL), while also preserving the environment. Analytical GREEnness (AGREE) software and the eco-scale (ES) metric were used to meticulously evaluate the ecological effects of the suggested UV methods^14,15^, while blue applicability grade index (BAGI) software and the Red Green Blue 12 (RGB12) model assessed their applicability (practicality) and whiteness, respectively^16,17^.

In conclusion, our suggested techniques offer significant advancements in terms of sustainability and practical applicability, along with a groundbreaking application in urine analysis, representing a valuable addition or practical alternative to all existing methodologies^10–12^.

## Experimental

### Materials and chemicals

- Pure MIR was kindly provided via Apex Pharma Company (Badr City, Egypt), while pure SIL was kindly provided via Eva Pharma Company (10th of Ramadan City, Egypt) with licensed purities of 99.86% ± 0.59 and 99.73% ± 0.86, in order.
- Magnesium sulfate (MgSO_4_), HPLC-grade ethanol, and methanol were acquired via Sigma-Aldrich, Egypt.
- Silotime-M 50 tablets, Intas Pharmaceuticals Ltd. (India); claimed from the community’s marketplace to contain 50 mg MIR and 8 mg SIL for each tablet; batch number (34/UA/2OI3).
- Healthy individuals provided urine samples, which were frozen until analysis.

### Apparatus

- The measurements were obtained using a Jasco UV-VIS spectrophotometer (dual-beam, V-360, Japan) equipped with Spectra Manager II software. The apparatus (Made by Jasco) was set to an optical scanning speed of 1000 nm.min^−1^ and a spectral slit width of 2 nm.
- Sonicator (Made by BANDELIN Electronic, Model RK 510 S, Germany).
- Benchtop centrifuge (Made by Centurion Scientifis, Model K241R, UK).

### Standard solutions

To create two separate working solutions of MIR and SIL with a concentration of 500 µg/mL, a standard stock solution of 1 mg/mL for each was prepared in ethanol. The same solvent was then used to dilute each stock solution (1 mg/mL) for preparing each working solution (500 µg/mL) in a volumetric flask (100 mL).

## General procedures

### Setup of calibration graphs

Two groups of 10-mL volumetric flasks were used to precisely separate aliquots of MIR and SIL standard (pure) solutions (500 µg/mL). The final concentrations of MIR (50–350) µg/mL and SIL (5-100) µg/mL were meticulously achieved by adding ethanol. The absorption spectra of the resulting concentrations were consecutively scanned and recorded on a computer, ranging from 200 to 400 nm, with ethanol primarily utilized as a blank.

#### IDW technique

For MIR, the absorbance values of MIR’s zero-order spectra were measured at λ_1_ (λ_max_, 250 nm) and λ_2_ (265 nm). Subsequently, the MIR’s absorbances at 265 nm were multiplied by the standard SIL’s equality factor (F_eq_) = A_250_/A_265_ = 0.816. For SIL, the absorbance values of SIL’s zero-order spectra were measured at λ_1_ (λ_max_, 268 nm) and λ_2_ (283 nm). After that, the SIL’s absorbances at 283 nm were multiplied by the standard MIR’s F_eq_ = A_268_/A_283_ = 4.096. Finally, the new absorbance values at λ_2_ (265 nm and 283 nm) were subtracted from the absorbances at λ_1_ (250 nm and 268 nm) for MIR and SIL, in order. Finally, the difference values (ΔA) at the aforesaid wavelengths were plotted against MIR’s and SIL’s corresponding concentrations (µg/mL), ranging from (50–350) µg/mL and (5–100) µg/mL, respectively, to create their calibration curves.

#### FSD technique

In this technique, MIR’s and SIL’s zero-order spectra at different concentrations were handled employing the spectrophotometer software. The software’s deconvolution algorithm was utilized to deconvolute the recorded spectra of MIR and SIL, with a full-width at half-maximum (FWHM) of 52. Finally, MIR’s and SIL’s deconvoluted amplitudes were measured at 254 nm and 273 nm for their corresponding concentrations (µg/mL), ranging from (50–350) µg/mL and (5–100) µg/mL, respectively, to construct their calibration curves.

### Techniques applications

#### Synthetic mixtures

Five laboratory mixes of MIR and SIL with varying complementary ratios were prepared by transferring the two substances’ aliquots (ranging from 1.8 to 6 mL for MIR and 0.8 to 1.8 mL for SIL) from their corresponding standard solutions (500 µg/mL) into a group of 10-mL volumetric flasks to appraise both analytical and validation specifics of the suggested methods. After transferring, each flask was diluted with ethanol and inverted repeatedly to ensure thorough mixing, resulting in concentration ranges of 90–300 µg/mL for MIR and 40–90 µg/mL for SIL. Finally, the regression equation for each proposed method was employed to quantify each component in every combination that was created in the laboratory.

#### Pharmaceutical formulation

To determine the quantities of MIR and SIL present in Silotime-M 50 tablets, the following steps were implemented: First, the average weight of 10 tablets was reckoned, followed by grinding these tablets. Second, an amount equal to one tablet that contains 50 mg MIR and 8 mg SIL was weighed and transferred, along with 20 mL ethanol, to a 50 mL volumetric flask. Third, the mixture was sonicated for 10 min and filtered after adding the final volume of ethanol. Fourth, the same solvent was used for further dilutions, resulting in a final tablet extract with 150 µg/mL of MIR and 24 µg/mL of SIL. Finally, the regression equation for each proposed method was employed to quantify MIR’s and SIL’s concentrations in their tablets.

#### Urine samples (SALLE procedure)

1000 µL portions of human urine were spiked with varying amounts of MIR and SIL working standard solutions, followed by vortex-mixing for a half minute. Afterward, a one-step SALLE method was carried out with one milliliter of 2 M MgSO_4_ and three milliliters of methanol. The samples were vortex-mixed for a half minute to extract the aforesaid drugs. At 4500 rpm, the solutions underwent a five-minute centrifugation to facilitate phase separation. The top organic phase (methanol) was carefully collected and handled via a continuous flow of nitrogen gas to thoroughly dry the methanol extract. The residues were reconstituted with three milliliters of ethanol and prepared for standard testing using the FSD method. The concentrations were then determined with precision using the associated regression parameters.

## Results and discussion

**Fig. 2:**
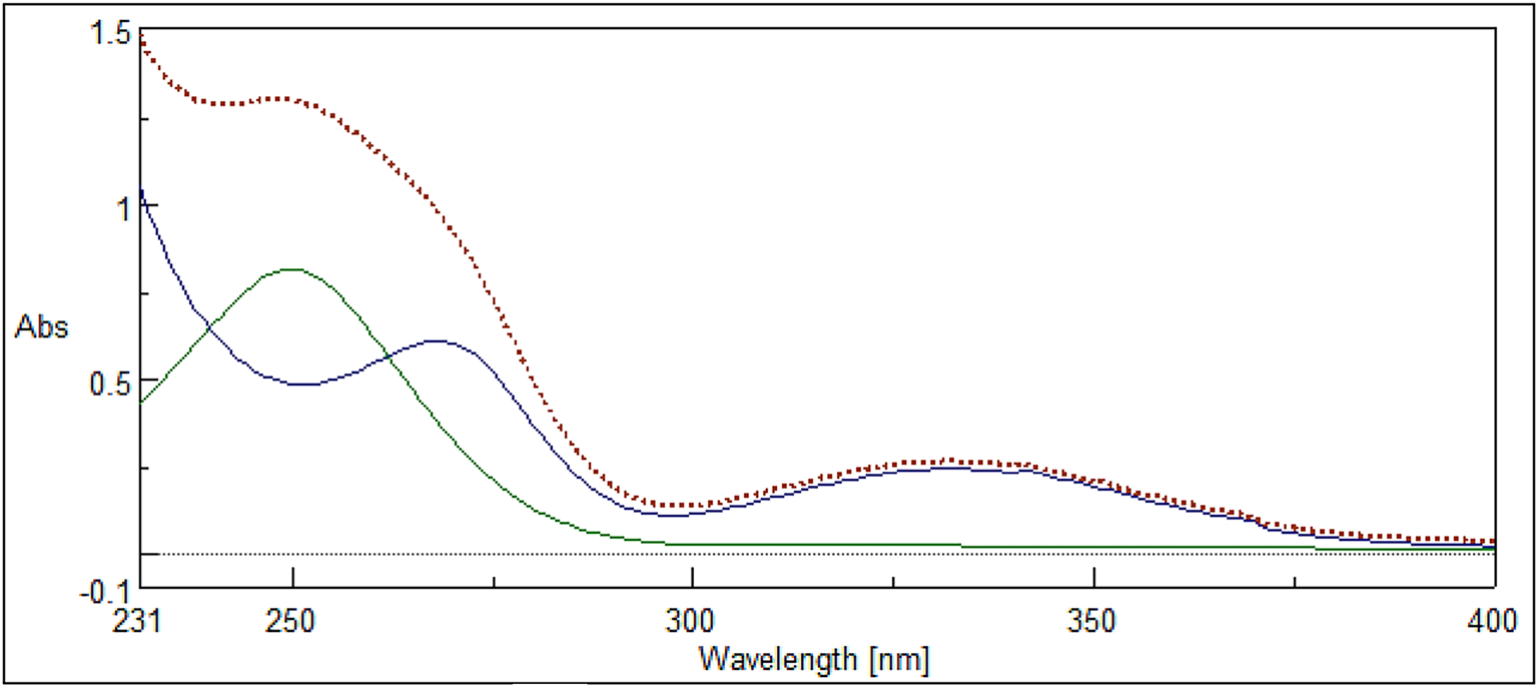
Zero-order absorption spectra of 250 µg/mL MIR (green spectrum) overlaid with 40 µg/mL SIL (blue spectrum) and their mix (dotted spectrum) using ethanol as a blank.

The advancement of analytical chemistry is vital for the development of new and effective methods for identifying and separating pharmaceutical compounds. However, this pursuit comes with potential risks that can pose a threat to both the environment and people. It is imperative that we continue to strive for economical, efficient, and safe procedures that prioritize the well-being of both our planet and its inhabitants. As a result, providing green tools for analysis with similar effective results has become a worldwide priority. Owing to the absence of reported UV methods capable of resolving the significant overlap between MIR and SIL signals (Fig. 2), particularly within the complex matrices of commercial tablets and human urine, it is crucial to establish environmentally friendly UV methods offering reliable analysis of these medications across diverse matrices, including pure powders, tablets, and urine samples.

### IDW technique

**Fig. 3:**
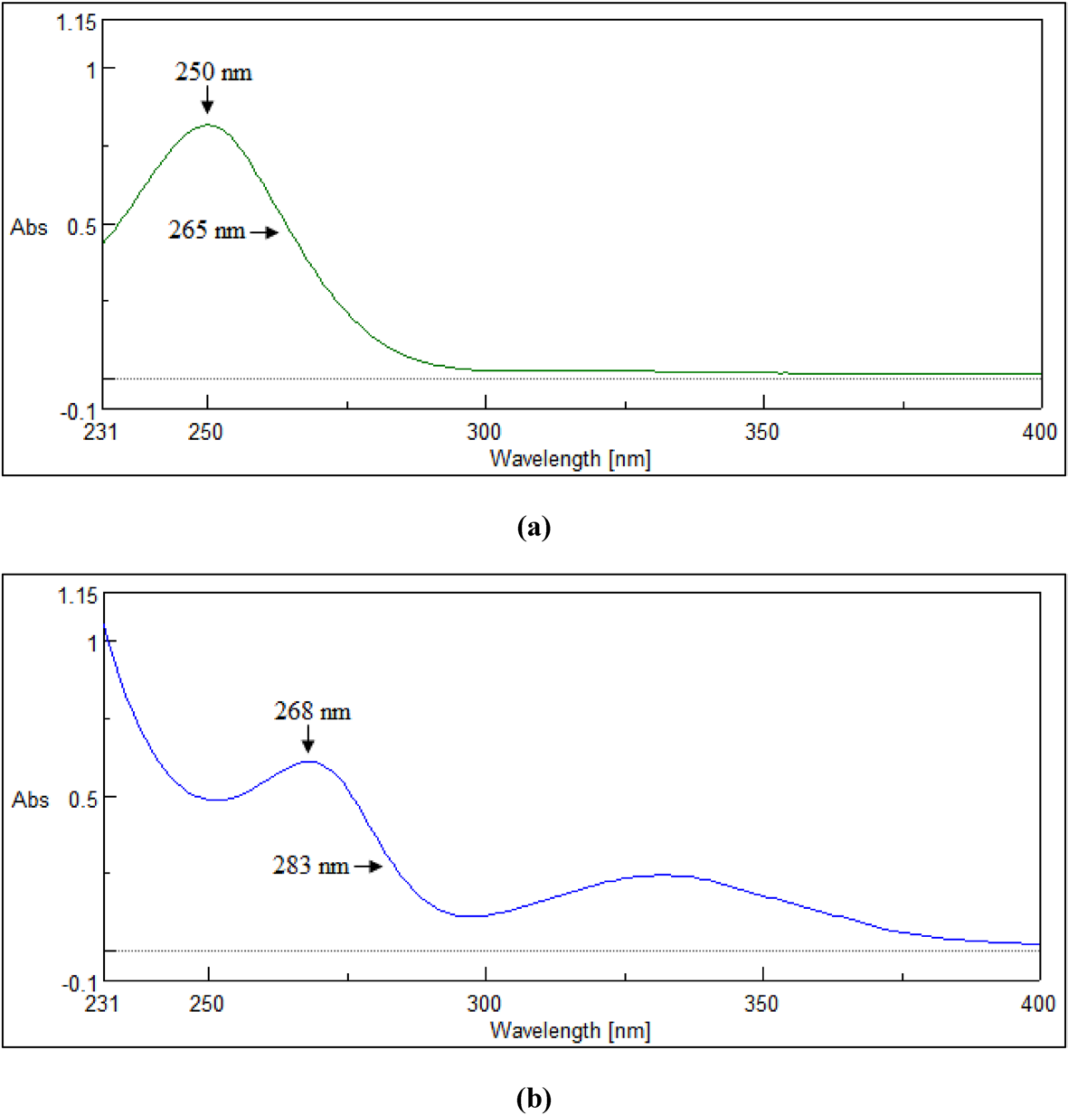
The chosen wavelengths for determination of (**a**) MIR (250 µg/mL) and (**b**) SIL (40 µg/mL) by the IDW technique.

When the traditional dual-wavelength (DW) approach is unsuccessful, this method (IDW) is applied^18,19^. In DW, two wavelength points are chosen to create a difference for one drug (X) and a zero difference for the second drug (Y), allowing drug X to be identified without interference from Y. However, this wasn’t applicable with MIR and SIL in their mixtures because, at the two precisely chosen wavelength points, 250 and 265 nm for MIR and 268 and 283 nm for SIL, there was a difference that wasn’t zero for any drug, hence the interfering influence remains present (Fig. 3). Eliminating the interfering element’s absorbance within two specific wavelengths by employing the F_eq_ is the guiding idea here.

As per the mathematical processing detailed in Sect. 3.1.1, the calculated difference values (ΔA) were plotted against MIR’s and SIL’s respective concentrations (µg/mL), ranging from (50–350) µg/mL and (5–100) µg/mL, in order, to create their calibration curves.

We optimized the selection of λ_2_ for each drug by conducting several trials across various wavelengths to find the λ that yielded the strongest linear relationship, reflected in the highest correlation coefficient. The F_eq_ was mainly utilized to level the responses (absorbances) of SIL (the interfering medication) at the aforesaid wavelengths (λ_1_ and λ_2_), where the responses of MIR were different and vice versa. This facilitates canceling the interfering drug via the subtraction step.

### FSD technique

This approach offers several advantages. Firstly, it is straightforward to implement and requires minimal computational resources. This makes it highly accessible and suitable for a wide range of applications. Secondly, it is affordable, as it eliminates the need for expensive and time-consuming separation techniques. Lastly, it is dependable, as it uses well-established computational methods and reliable spectral analysis techniques to achieve accurate results^20^. The final outcome of this technique involves canceling the Y-axis datum to highlight the X-axis datum, which results in a higher resolution of the strongly overlapped signals.

**Fig. 4:**
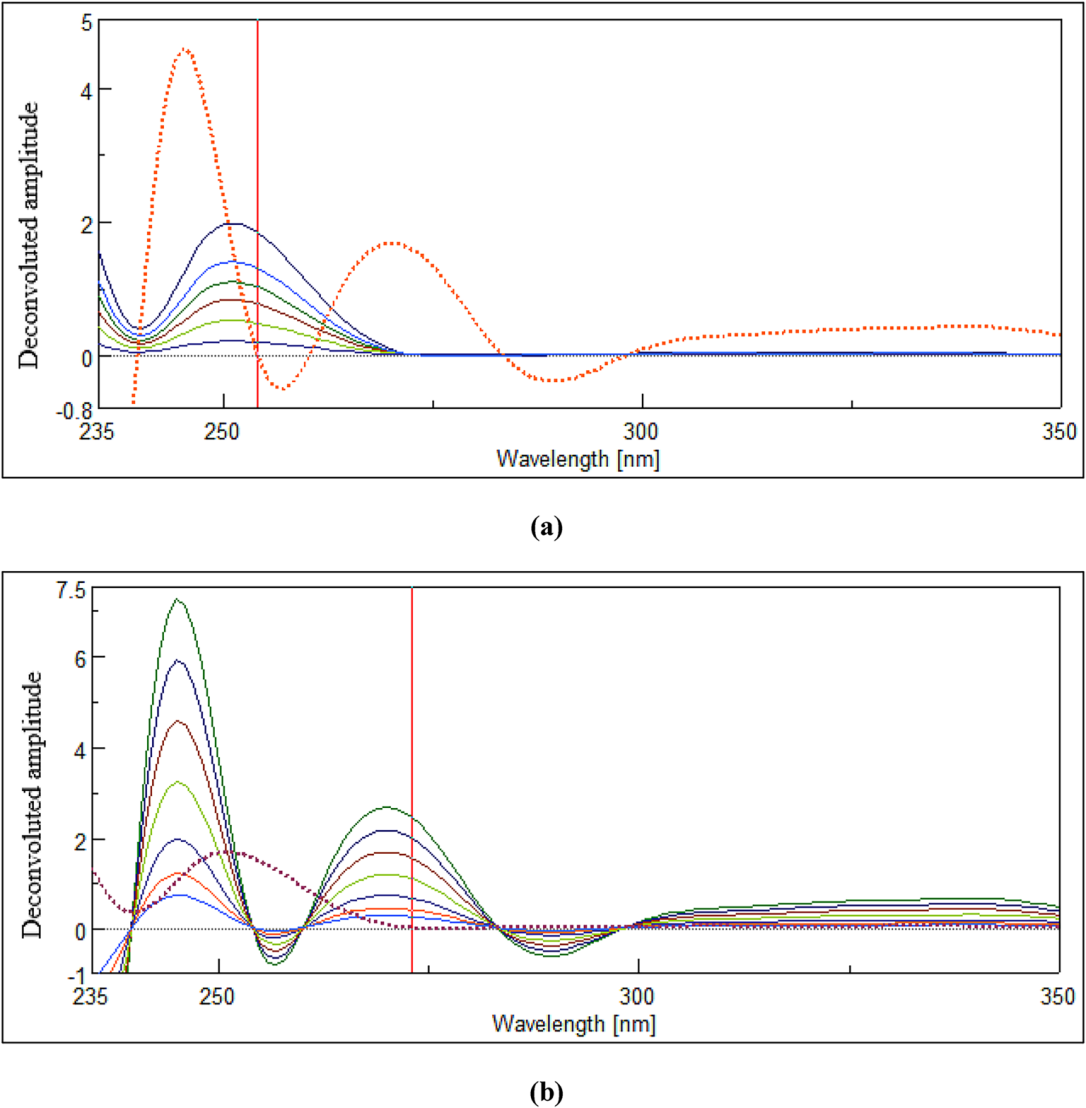
Deconvoluted spectra of (**a**) MIR (50–350) µg/mL computed at 254 nm, where a zero-crossing point of SIL deconvoluted spectrum (dotted spectrum) and (**b**) SIL (5–100) µg/mL computed at 273 nm, where no intrusion from MIR deconvoluted spectrum (dotted spectrum).

Here, the process was carried out by adjusting the FWHM icon, designed within the spectrophotometer software, to a value of 52 to achieve complete cancellation of the spectral contribution of one drug at the peak wavelength of the other. We tested other FWHM values (both lower and higher), but found that all values other than 52 resulted in unacceptable levels of interference between the studied drugs’ signals. Hence, MIR’s deconvoluted amplitudes were measured at 254 nm, where SIL was zero-crossing (no interfering) (Fig. 4a), while SIL’s deconvoluted amplitudes were measured at 273 nm, where MIR was zero-crossing (no interfering) (Fig. 4b). Employing the corresponding calibrations (Sect. 3.1.2), amounts of MIR and SIL were computed in their mixtures.

## Validation of methods

The suggested methods were subject to validation in accordance with ICH criteria^21^.

### Linearity

As per the aforesaid experimental settings, different concentrated solutions of MIR and SIL, respectively, ranging from 50 to 350 µg/mL and 5 to 100 µg/mL, were analyzed to evaluate linearity. Three assessments were made for each concentration. Table 1 lists the linear ranges for each of the recommended techniques.

**Table 1:** Assay parameters and validation data of the suggested UV techniques for MIR and SIL determination.

Techniques	IDW		FSD	
Drugs	MIR	SIL	MIR	SIL
Parameters				
Wavelength (nm)	ΔA (250–265)	ΔA (268–283)	254	273
Linearity range (µg/mL)	50–350	5-100	50–350	5–100
Correlation coefficient (r)	0.9998	0.9999	0.9998	0.9999
Slope	0.0018	0.0112	0.0054	0.0226
Intercept	-0.0232	0.0955	-0.0715	0.1718
SD of intercept	0.0029	0.0032	0.0096	0.0079
LOD (µg/mL)	5.32	0.94	5.87	1.15
LOQ (µg/mL)	16.11	2.86	17.78	3.49
Accuracy (R% ± SD) ^a^	100.52 ± 0.71	98.95 ± 0.91	100.29 ± 0.83	99.56 ± 1.11
Precision				
Repeatability (RSD%) ^b^	0.88	0.79	0.96	0.80
Intermediate precision (RSD%)^c^	0.90	0.84	1.04	0.92

^a^Mean of three determinations.

^b^The repeatability; relative standard deviation of (100, 200, and 300 µg/mL) of MIR and (20, 50, and 80 µg/mL) of SIL in triplicate within the day using the suggested UV techniques.

^c^The intermediate precision; relative standard deviation of (100, 200, and 300 µg/mL) of MIR and (20, 50, and 80 µg/mL) of SIL in triplicate on three consecutive days using the suggested UV techniques.

### Accuracy

The recommended techniques’ accuracy was verified by calculating the average recovery of three distinct concentrations (estimated three times). MIR’s concentrations were 100, 200, and 300 µg/mL, while SIL’s concentrations were 20, 50, and 80 µg/mL. Table 1 illustrates the outcomes.

### Precision

The proposed techniques were tested for both intraday and interday precisions by running three independent experiments with three distinct concentrations (estimated three times) over one day or three days (successive days). The levels of MIR were 100, 200, and 300 µg/mL, while the levels of SIL were 20, 50, and 80 µg/mL. At the two precision levels, relative standard deviation (RSD) outcomes were acceptable, as listed in (Table 1).

### Limit of detection (LOD) and quantification (LOQ)

The procedures that were previously discussed were subjected to the following computations, which measured both LOQ and LOD to evaluate the methods’ sensitivity.

LOQ = 10xSD/Slope; LOD = 3.3xSD/Slope; SD is the standard deviation.

The obtained results ascertain the sensitivity of the recommended procedures (Table 1).

### Specificity

Specificity was confirmed by assessing multiple synthetic mixtures incorporating the examined medicines in varied ratios inside their linear limits. The ratios, presented in Table 2, were carefully selected to reflect the dosage form ratio, represent potential variations in drugs’ concentrations, and challenge the methods with significant excesses of one drug over the other. Table 2 provides excellent results that ascertain the specificity of the offered procedures.

**Table 2:** Determination of MIR and SIL in their laboratory mixtures by the suggested UV techniques.

Techniques		IDW		FSD	
Drugs		MIR	SIL	MIR	SIL
Concentration (ug/mL)					
MIR	SIL	Recovery% ^a^			
90	90	100.36	99.08	98.55	100.34
100	50	100.96	98.68	100.67	100.80
300	60	99.61	99.49	100.52	101.72
200	50	100.09	98.98	101.08	101.09
250 ^b^	40 ^b^	99.79	98.87	100.05	99.51
Mean ± SD		100.16 ± 0.53	99.02 ± 0.30	100.17 ± 0.98	100.69 ± 0.83

^a^Mean of three determinations.

^b^The same concentration ratio in Silotime-M 50 tablets.

## Techniques applications

### Pharmaceutical formulation

MIR and SIL in the Silotime-M 50 tablets were carefully analyzed using the approaches mentioned before. Accurate and reliable results were achieved for all methods, as evidenced in (Table 3), definitively proving that the pharmaceutical additives did not disrupt the drug analysis.

**Table 3:** Determination of MIR and SIL in Silotime-M 50 tablets using the suggested UV techniques and application of standard addition protocol.

Techniques	IDW		FSD	
Drugs	MIR	SIL	MIR	SIL
Silotime-M 50 ^a^	100.61 ± 0.76	99.27 ± 1.02	101.01 ± 1.32	100.50 ± 1.20
Found % ± SD ^b^				
Standard addition	100.28 ± 1.06	99.63 ± 0.75	100.87 ± 1.10	100.71 ± 0.82
Pure found% ± SD ^c^				

^a^ Silotime-M 50 tablets; claimed to contain 50 mg MIR and 8 mg SIL per tablet; batch number (34/UA/2OI3).

^b^ Mean of three determinations.

^c^ Mean of five determinations.

### Urine samples (SALLE procedure)

**Fig. 5:**
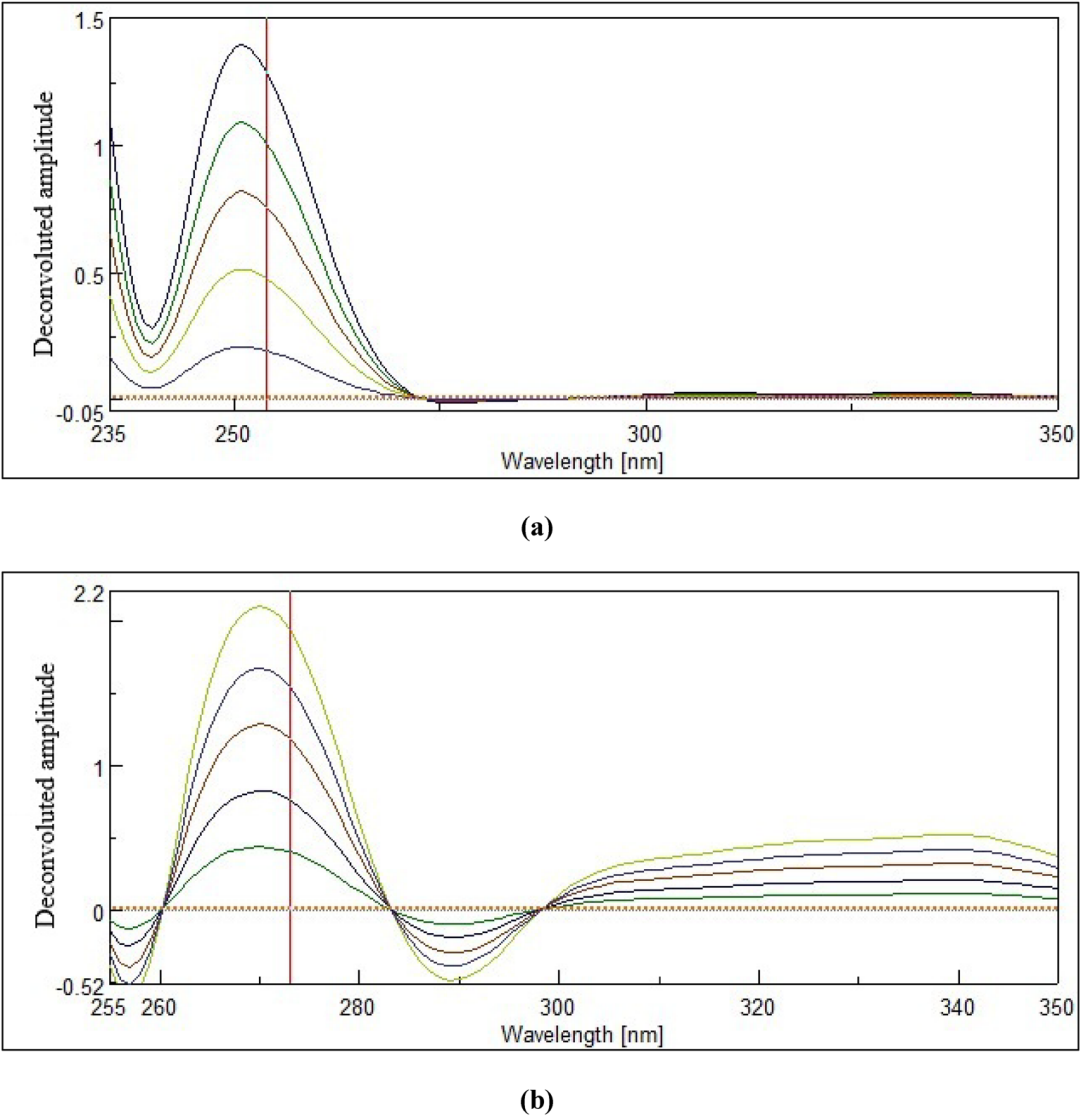
Deconvoluted spectra of (**a**) MIR (60–300) µg/mL in urine (computed at 254 nm) and (**b**) SIL (10–50) µg/mL in urine (computed at 273 nm), where no intrusion from the blank urine deconvoluted spectrum (dotted spectrum).

The prior study of MIR pharmacokinetics showed that about 55% of a 50-mg dose is excreted as MIR in urine^22^, while the prior study of SIL showed that about 33.50% of an 8-mg dose is excreted as SIL in urine^23^. The studied drugs were extracted from urine samples using the SALLE process. This involved adding salts to the aqueous medium and using an organic solvent that is miscible with water. As a result of this procedure, the two liquids became less soluble, leading to the formation of two distinct layers from which the essential analyte was extracted into the organic layer^13^. The selected drugs were extracted into methanol with the assistance of MgSO_4_. SALLE offers a range of merits, such as high analyte recovery, rapid sample processing, minimal experimental costs, and ease of operation. This extraction process does not inherently necessitate evaporation. However, following extraction, we employed a continuous flow of nitrogen gas as an optional step to facilitate the removal of residual solvent and concentrate the drug extract, thereby improving the sensitivity of subsequent analyses. Also, the use of environmentally benign solvents in this process ensures minimal impact. The method’s exceptional sensitivity enabled precise measurement of MIR and SIL in urine without matrix interference, as demonstrated in (Fig. 5). Table 4’s outcomes highlight excellent recovery percentages, indicating the immense potential of this system in evaluating the studied drugs clinically in biofluids. Validation parameters (Table 4) were calculated according to ICH criteria^21^.

**Table 4:** Validation parameters for quantification of MIR and SIL by FSD technique in spiked human urine.

Drugs	MIR	SIL
Parameters		
Linearity range (µg/mL)	60–300	10–50
Correlation coefficient (r)	0.9998	0.9997
Slope	0.0045	0.0385
Intercept	−0.0821	0.0028
SD of intercept	0.0092	0.0167
LOD (µg/mL)	6.75	1.43
LOQ (µg/mL)	20.44	4.34
Accuracy (R% ± SD)*	101.07 ± 1.36	98.89 ± 1.42
Precision		
Repeatability (RSD%)	1.19	1.24
Intermediate precision (RSD%)	1.25	1.51

*Mean of five determinations.

## Greenness and whiteness evaluations

To appraise how eco-friendly the suggested techniques are, we selected AGREE and ES metrics. AGREE is a free software package utilized to create a 12-segment circular pictogram embracing every one of the twelve facets of GAC^14,24,25^. Depending on how the analysis approach influences the surroundings, each segment’s color varies from deep green (lowest ecological impact) to deep red (highest ecological impact), with a valuation score in AGREE’s pictogram center. Table 5’s AGREE pictograms for both suggested and reported^10−12^ techniques with valuation scores disclose the suggested techniques’ preeminence from a greenness standpoint.

**Table 5:** Greenness assessment of the suggested and reported^10−12^ techniques by Eco-Scale and AGREE tools.

Tools	Suggested techniques	Reported technique^10^	Reported technique^11^	Reported technique^12^
Eco-scale				
Reagents				
*Ethanol*	4	-	-	-
*Ammonium acetate buffer*	-	0	-	-
*Phosphate buffer*	-	-	0	-
*Acetonitrile*	-	8	-	-
*Methanol*	6	6	12	12
*Magnesium sulfate*	0	-	-	-
Instrument				
*Energy consumption*	0	1	1	0
*Occupational Hazard*	0	0	0	0
*Waste*	3	5	5	3
Total PPs	13	20	18	15
Analytical eco-scale total score^a, b^	87	80	82	85
Comment	Excellent green analysis	Excellent green analysis	Excellent green analysis	Excellent green analysis
AGREE				

^a^ Analytical eco-scale total score = 100- total penalty points.

^b^ If the score is > 75, it signifies excellent green analysis.

If the score is > 50, it signifies acceptable green analysis.

If the score is < 50, it signifies inadequate green analysis.

Also, Table 5 reveals that the suggested techniques have a superior greenness score when taking into consideration the ES metric, as determined by calculating the ES scores and penalty points for the suggested and reported^10,11,12^ techniques. The aforesaid metrics (AGREE and ES), in summary, display consistent results (Table 5).

**Fig. 6:**
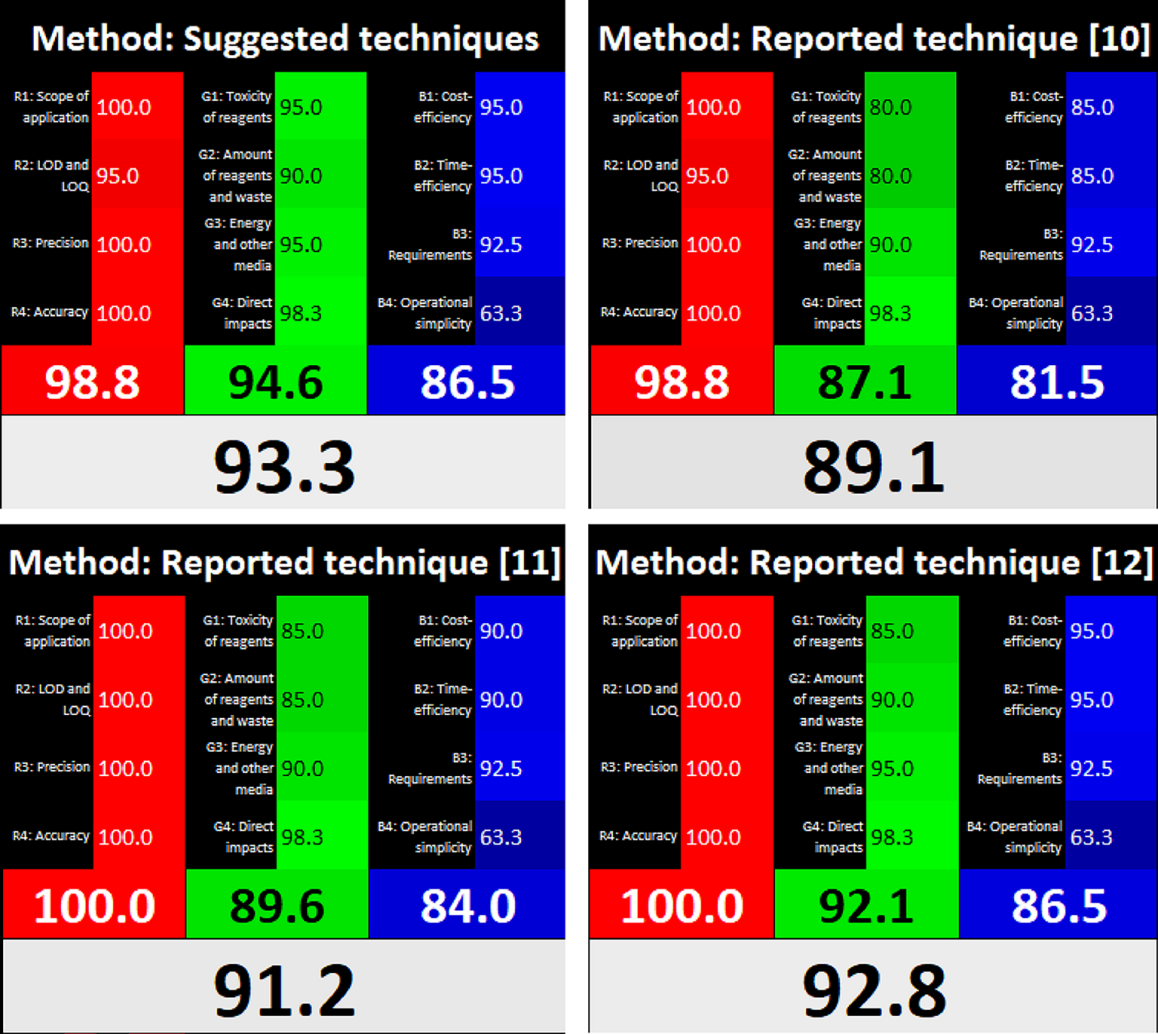
Whiteness evaluation of the suggested and reported^10−12^ techniques by the RGB12 model.

To appraise “whiteness,” the suggested techniques and the earlier methods^10,11,12^ were tested using the RGB12 model^17^. This model consists of twelve algorithms, run in Excel, that are segmented into categories based on blue, green, and red colors. A comparative analysis based on this evaluation, as depicted in Fig. 6, demonstrates the superior “whiteness” performance of the suggested techniques.

## Blueness (practicality) evaluation

**Fig. 7:**
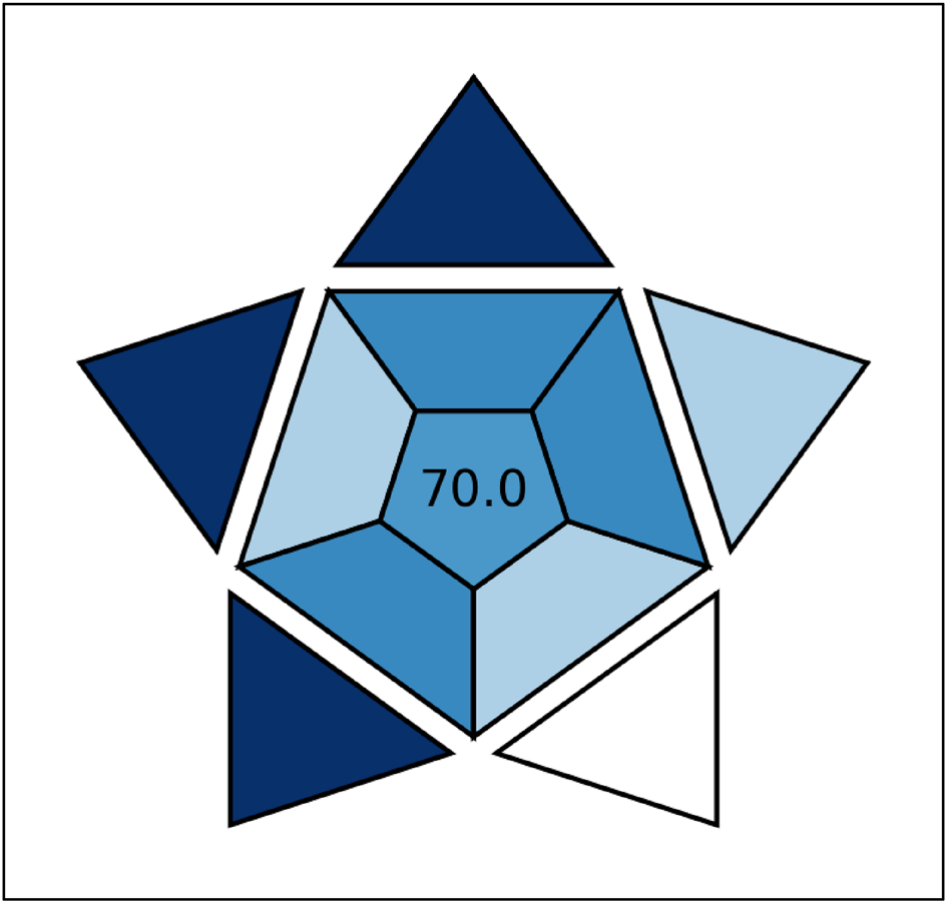
Blueness assessment of the suggested techniques by the BAGI tool.

To appraise the suggested techniques’ applicability (practical considerations), we selected the recently introduced ‘‘BAGI’’ metric. The BAGI considers ten criteria to produce a 10-segment asteroid-shaped pictogram with a central valuation score for testing the analytical approach’s efficacy and practicality^16,26^. To be considered “practical,” the technique should have a valuation score of more than 60. The validation principles of BAGI are analysis type, instrumentation, number of analytes, needs for sample preparation, amount of sample, sample throughput, samples analyzed apiece hour, degree of automation, preconcentration needs, and reagents/materials required. Depending on the aforementioned principles, each segment’s color varies from dark blue (for high compliance, applicability, or fit-for-purpose) to white (for non-compliance). Our UV techniques achieved a high BAGI score of 70.0, signifying the practicality or applicability of the suggested techniques (Fig. 7).

## Statistical analysis

With the use of the student t-test and F-test, we were able to provide statistically significant comparisons of recoveries between each recommended method and reported approach^10^. Based on the calculated data (Table 6), there were no statistically significant differences observed between any of the described methods and the reported approach. This was evidenced by the computed student t-test and F-test values, both of which were found to be smaller than their respective critical (theoretical) values.

**Table 6:** Statistical comparison of the findings revealed by the suggested techniques and the reported technique^10^ for the analysis of MIR and SIL in their Silotime-M 50 tablets.

Drugs	MIR			SIL		
Techniques	IDW	FSD	Reported technique	IDW	FSD	Reported technique
Parameters						
Mean	100.55	99.64	99.86	100.42	99.84	100.86
SD	1.02	0.87	0.74	0.82	1.04	1.12
N	5	5	5	5	5	5
Variance	1.04	0.76	0.55	0.67	1.08	1.25
Student^’^s *t*-test (2.31)*	1.22	0.43	–	0.71	1.49	–
F-value (6.39)*	1.90	0.72	–	1.87	1.16	–

*The parentheses contain the corresponding theoretical *t* and F values at (*P* = 0.05).

## Conclusion

The presented UV techniques are characterized by their simplicity, precision, and selectivity. These innovative techniques offer the distinct advantage of requiring minimal mathematical adjustments to MIR’s and SIL’s zero-order spectra, making them efficient and straightforward. With high sensitivity, our techniques accurately distinguished MIR and SIL from each other in their different mixtures, in addition to determining the selected drugs in urine samples by employing the SALLE technique. The SALLE method offered an affordable extraction for the selected drugs with fewer steps and enhanced precision, sensitivity, accuracy, and full selectivity. Evaluation of our techniques using ES, AGREE, and RGB12 metrics guarantees environment and analyst protection against hazardous exposure to chemicals, while evaluation using the BAGI tool guarantees the applicability of the suggested techniques. Accordingly, our techniques can be seamlessly integrated into the pharmaceutical and medicinal compounds industry for routine QC work.

## Data Availability

All data generated or analyzed during this study are included in this published article.
